# Psychotic Symptoms Associated With a Frontoparietal Arachnoid Cyst: The Role of Neuroimaging Studies in First-Episode Psychosis

**DOI:** 10.7759/cureus.31652

**Published:** 2022-11-18

**Authors:** Antonio Melo, Amilcar S Santos

**Affiliations:** 1 Psychiatry, Hospital de Vila Franca de Xira, Vila Franca de Xira, PRT; 2 Faculdade de Ciências Médicas, Nova Medical School, Lisbon, PRT

**Keywords:** neuro-imaging, treatment resistant psychosis, first episode psychosis, treatment-resistant psychosis, huge arachnoid cyst

## Abstract

Arachnoid cysts, although usually asymptomatic, can be associated with psychiatric symptoms, including delusions and hallucinations. The role of neuroimaging findings, particularly arachnoid cysts, and their influence on psychiatric symptoms is still controversial and debated. We present the case of a 56-year-old male who sought medical help for his psychotic symptoms, mainly paranoid delusions, and auditory and tactile hallucinations. Brain imaging studies at the time of admission revealed a large left frontoparietal arachnoid cyst. The patient was then started on psychiatric medication but did not show any sign of clinical improvement. After discussing the case with the patient´s neurosurgeon, it was decided to submit the patient to drainage of his arachnoid cyst. The patient was reevaluated two weeks after the procedure showing significant clinical improvement, particularly in his positive psychotic symptoms. The rapid improvement of the psychotic symptoms after removing the cyst suggests that, at least in part, the mass was contributing to the symptoms presented.

## Introduction

An arachnoid cyst is a collection of cerebrospinal fluid that accounts for approximately 1% of all space-occupying non-traumatic intracranial lesions [1]. Although sometimes asymptomatic, arachnoid cysts can be associated with psychiatric symptoms, such as psychosis, attention deficit, depression, insomnia or irritability. These symptoms may or may not improve after surgical decompression [2].

Current medical literature states that when assessing a patient during his first psychotic episode, a careful assessment should be undertaken, which might include imaging studies [3]. However, the role of neuroimaging findings, particularly arachnoid cysts, and their influence on psychiatric symptoms is still not fully understood.

## Case presentation

A 56-year-old male presented to the emergency room with psychotic symptoms, mainly paranoid delusions, delusions of jealousy and auditory and tactile hallucinations. He complained that his upstairs neighbor was controlling his mind through the use of radio waves. According to the patient, this neighbor was trying to apply those radio waves to weaken him in order to seduce his wife. The patient said that he could feel the radio waves as a sensation of heat throughout his body. Moreover, the intensity of the waves decreased when he changed position in bed (from left lateral decubitus to the supine position) or covered his head with a pillow. He also complained of hearing the voice of his neighbor, whispering in his ear, describing how he was going to seduce and murder his wife. He was also convinced that the radiation was making him feel nauseous. His speech was accelerated and disorganized; he was experiencing severe anxiety and insomnia. Overall, he scored 139 out of 210 on the Positive and Negative Syndrome Scale (PANSS), mainly due to positive symptoms (42/49).

According to the patient's wife, these behavioral changes had begun six months prior to the visit to the emergency room. She told us that her husband had grown increasingly paranoid and violent, constantly worrying about the referred upstairs neighbor. She feared her husband, as there had been multiple episodes of violence and domestic abuse, which only began after the behavioral changes. According to the wife, the upstairs apartment was vacant, and the last occupant had left months before the start of these symptoms.

After searching through the patient's medical records, we discovered that seven years prior to this visit, he was suffering from frequent episodes of lipothymia. At that time, Brain Computed Axial Tomography (CT) and Magnetic Resonance Imaging (MRI) showed a large left frontal arachnoid cyst. After consulting a neurosurgeon, the patient underwent left frontal trepanation and drainage of the cyst. The patient showed full remission of his symptoms at discharge, and the previously detected cyst was no longer observable on post-surgical imaging scans. During the episode, the patient did not present any psychotic symptoms.

He had his last neurosurgery consultation five years after the procedure, being clinically stable at the time. Besides this intervention, the patient also had a history of mild depression, which was controlled with sertraline 50mg daily. There was no further psychiatric history other than what was previously mentioned. There was also no relevant family history of psychiatric disease.

**Figure 1:**
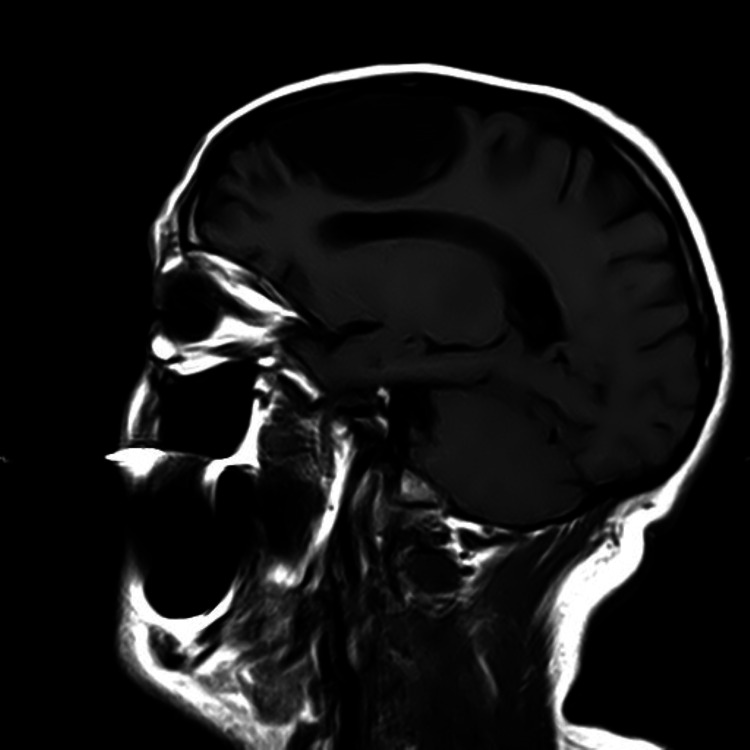
Magnetic Resonance Imaging showing a large left frontal arachnoid cyst - Sagittal view.

**Figure 2:**
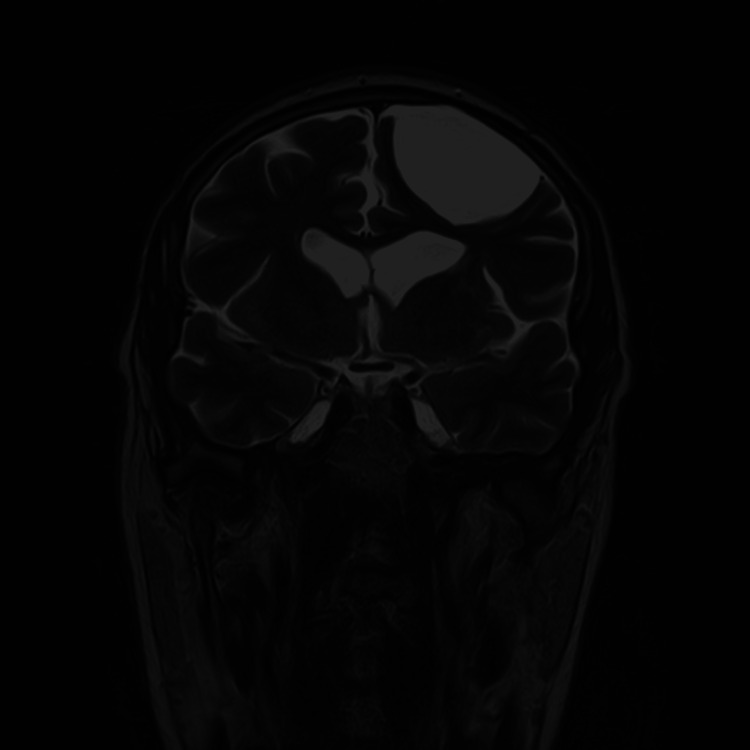
Magnetic Resonance Imaging showing a large left frontal arachnoid cyst - Coronal view.

**Figure 3:**
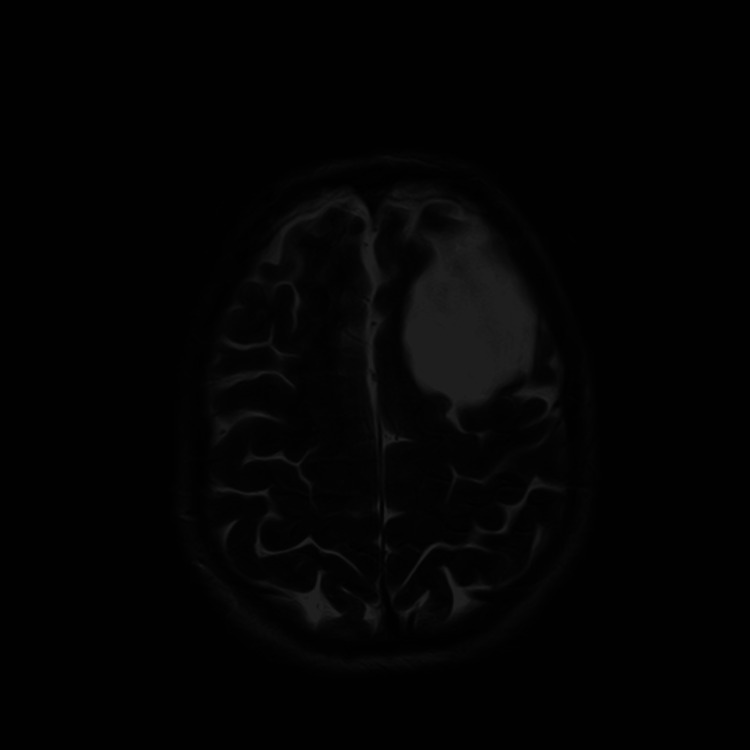
Magnetic Resonance Imaging showing a large left frontal arachnoid cyst - Axial view.

At the emergency room, after psychiatric examination, the patient was hospitalized for further care. CT and MRI were undertaken, revealing regrowth of his large left frontal arachnoid cyst (Figures 1, 2, 3). Laboratory analysis of the patient's blood showed no relevant clinical findings, and his EKG was normal. The patient was also started on psychiatric medication (olanzapine 20mg daily; valproic acid 1000mg daily and diazepam 15mg daily), but did not show any sign of clinical improvement after two weeks of therapy.

**Figure 4:**
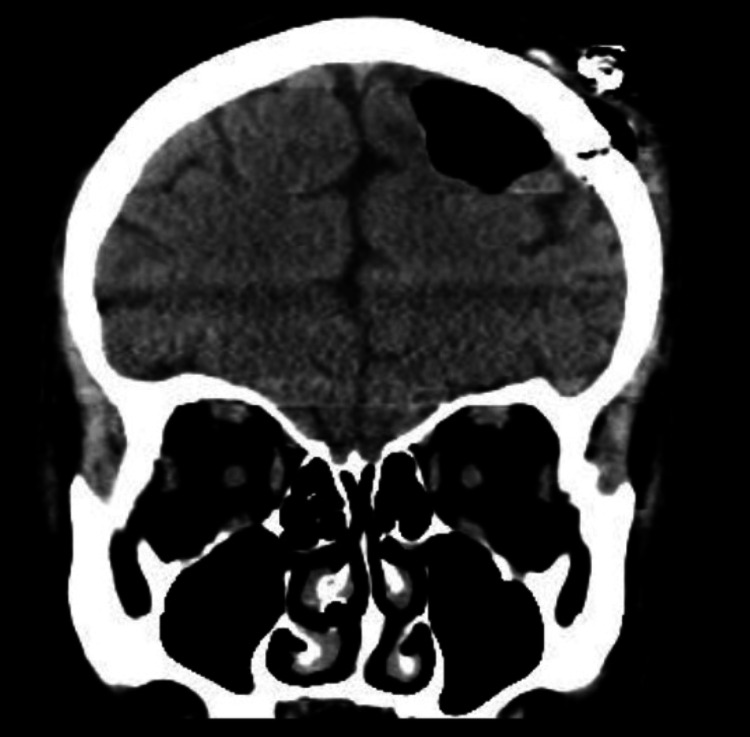
Post-surgical Computed Tomography scan, showing complete removal of the cyst - Coronal view.

**Figure 5:**
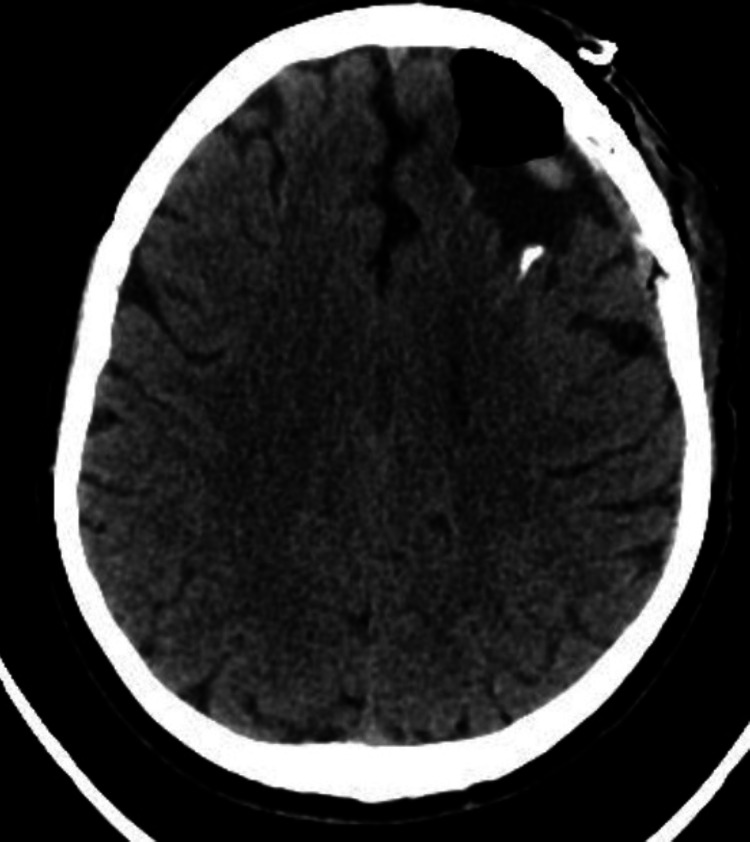
Post-surgical Computed Tomography scan, showing complete removal of the cyst - Axial view.

After discussing the case with the patient's neurosurgeon, it was decided to submit the patient to new drainage of his arachnoid cyst. After the procedure, subsequent imaging studies showed complete removal of the cyst (Figures 4, 5).

The patient was reevaluated two weeks after the procedure showing significant clinical improvement, particularly in his positive psychotic symptoms. He no longer felt threatened by his neighbors. He even showed some insight into his condition: he told his physician that the sensation of heat running through his body, which was previously attributed to the radiation emitted by his neighbor, was probably just a product of his imagination, as he no longer felt it. Overall, the patient scored 41 out of 210 on the PANSS scale (with a 13 out of 49 on his positive symptoms), which was a significant improvement compared to his previous evaluation.

The patient was reevaluated two months after being discharged in a follow-up consult. He had discontinued all medication (against the doctors' recommendations) and scored a 40 on the PANSS scale. He maintained the delusional belief that he was being persecuted by someone (a person that the patient now claimed had been his neighbor, but no longer lived in his building). However, he was functional and able to maintain his daily routines. He distanced himself from this persecutory delusion, stating that this person no longer bothered him or his wife. The wife was also privately interviewed and ascertained her husband had improved and was no longer aggressive towards her. Other follow-up visits were scheduled. 

## Discussion

We should start by noting that this patient presents clinical findings suggesting an organic cause of his psychotic symptoms. Firstly, the patient had no previous history of psychotic symptoms. His psychiatric history included only mild depressive symptoms seven years before this episode. Inaugural functional psychotic episodes do not usually occur at such an advanced age [4]. The patient had no relevant psychiatric family history either.

However, it is, of course, impossible to state that the psychotic symptoms were fully caused by the arachnoid cyst (either due to physical compression, neurochemical imbalances or neurophysiological changes). The patient was under medical treatment for only a short period of time (14 days) before being submitted to surgical treatment. It is possible that his clinical presentation would have resolved with medical treatment alone.

However, the rapid improvement of the psychotic symptoms after removing the cyst (mainly the tactile hallucinations) suggests that, at least in part, the mass may have contributed to the symptoms presented. This fact raises our awareness regarding the role of neuroimaging studies and possible surgical indications in patients with psychotic symptoms, mainly in their first formal clinical presentation or first-episode psychosis. To this day, the usefulness of imaging studies in first-episode psychosis is still being challenged [4,5]; many neurosurgeons do not even consider surgery on patients presenting exclusively with psychiatric symptoms. As such, examining patients as a whole and reviewing their medical history may help decide the best course of treatment when presenting with both structural brain changes (as an arachnoid cyst) and psychotic symptoms.

This approach to clinical psychiatry includes the perspectives of different medical specialties and scientific areas (in this case, neurology/neuroimaging, neurosurgery and psychiatry). This can be considered a multidisciplinary integrative model for approaching mental disorders [6]. Finally, it is also important to state that there are several isolated cases in the medical literature of patients showing some improvement after removing large arachnoid cysts [7,8]. 

## Conclusions

In conclusion, patients presenting with first-episode psychosis and a concomitant arachnoid cyst may benefit from removal of said brain mass. However, the mechanism by which the mass may contribute to the psychiatric symptoms is not fully understood. As such, further research is needed, particularly studies that attempt to elucidate the specific biological mechanisms by which a large brain mass or cyst might contribute to psychiatric symptoms, whether it is due to compression, chemical imbalance, etc. Presently, one cannot know for sure if removal of an arachnoid cyst in first-episode psychosis is beneficial for the patient, and therefore each case must be considered individually.
